# Clinical Practice Patterns in Bone Health Assessment and Management in Endogenous Cushing's Syndrome

**DOI:** 10.1111/cen.70085

**Published:** 2025-12-21

**Authors:** Preeshila Behary, Dinushan Raveendran, Ramesh Nair, Aimee Di Marco, Debbie Papadopoulou, Florian Wernig, Ali Abbara, Karim Meeran, Niamh Martin, Jeremy Cox, Alexander N. Comninos

**Affiliations:** ^1^ Section of Endocrinology & Investigative Medicine Imperial College London London UK; ^2^ Endocrine Bone Unit Imperial College NHS Trust London UK; ^3^ Department of Endocrinology Imperial College NHS Trust London UK; ^4^ Department of Neurosurgery Imperial College NHS Trust London UK; ^5^ Department of Endocrine Surgery Imperial College NHS Trust London UK

**Keywords:** bone health, bone mineral density (BMD), calcium, Cushing's Syndrome, fragility fractures, osteoporosis, vitamin D

## Abstract

**Objective:**

Skeletal fragility is a common complication of endogenous Cushing's Syndrome (CS), although specific guidelines for managing bone health are lacking. This study aimed to assess clinicians' current engagement with bone health assessment and management in patients with endogenous CS.

**Design:**

Retrospective‐cohort design.

**Patients:**

Seventy‐nine patients with confirmed endogenous CS, treated at a tertiary endocrine centre.

**Measurements:**

The frequency of bone health assessment, evidenced by vitamin D measurement, and bone health management, evidenced by a composite outcome of calcium and/or vitamin D optimisation and/or initiation of bone‐protective agents, was recorded. Changes in bone mineral density (BMD), measured by Dual‐energy X‐ray absorptiometry (DEXA) and fracture prevalence were assessed pre‐ and post‐CS treatment.

**Results:**

Vitamin D was measured in only 43% (34/79), and bone health was managed in only 39.2% (31/79). BMD was assessed in 44.3% (35/79) during active CS; of these, 22.9% had osteoporosis. Improved BMD was observed within a year of CS remission. Fractures occurred in 17.7% (14/79) within 2 years of CS diagnosis, and 12 additional fractures occurred during follow‐up despite CS remission. Treatment with bone‐protective agents expedited recovery with a significant increase in lumbar spine BMD, compared to those not treated.

**Conclusions:**

Our data demonstrate that skeletal impairment and fragility fractures are highly prevalent in endogenous CS, and fracture risk may persist despite remission. However, currently, bone health is inadequately assessed and managed. These findings identify an urgent need for improved awareness, assessment, and management of bone‐health in this high‐risk population and call for specific evidence‐based practice guidelines.

## 1 Introduction

Endogenous Cushing's Syndrome (CS), characterised by abnormally high cortisol levels, has an annual incidence of approximately 3.2 cases per million [1]. The aetiology of endogenous CS includes adrenocorticotropic hormone (ACTH)‐secreting pituitary adenoma (Cushing's disease), ectopic ACTH‐producing tumours, and cortisol‐secreting adrenocortical tumours [2]. Skeletal impairment is a frequent but frequently overlooked complication of endogenous CS, with the prevalence of osteoporosis reported to be 22%−57% [2], as assessed by Dual‐energy X‐ray absorptiometry (DEXA).

Hypercortisolism disrupts the development, function, and survival of osteoblasts while promoting osteoclastic activity [2], ultimately resulting in reduced bone mineral density (BMD) by up to 20% [3] and degraded bone microarchitecture [4]. In fact, bone quality can be impacted even when BMD seems normal [5]. In addition, impaired intestinal and renal calcium handling and disrupted vitamin D metabolism in CS may exacerbate bone loss [6]. Consequently, fragility fractures are common, present in nearly half of CS patients [7].

Since endogenous CS affects trabecular bone more markedly than cortical bone [8], vertebral fractures are frequently observed. The reported prevalence of vertebral fractures is up to 76% in endogenous CS [9, 10], with up to 85% affecting multiple vertebrae [9]. These fractures significantly reduce quality of life due to persistent back pain, functional limitation, spinal deformities, and increase the risk of further fractures [11]. Moreover, fragility fractures can be the presenting feature of otherwise silent CS [6], with a threefold increased fracture risk 2−3 years preceding CS diagnosis [12, 13]. Therefore, prompt identification and management of bone health in endogenous CS is of paramount importance alongside management of underlying CS.

Remission of hypercortisolism, achieved by surgical or medical treatment, has been shown to induce bone recovery in prospective studies. A rise in serum osteocalcin up to fourfold has been observed within 1 year of biochemical remission [14], indicating increased osteoblastic function. Moreover, BMD improved in all skeletal compartments within 2 years [14, 15] but most markedly in the lumbar spine [16, 17]. In keeping with this, reduced fracture risk has been described following remission of endogenous CS [13, 18]. Spontaneous recovery of bone loss following the restoration of normocortisolaemia is therefore possible. However, complete bone recovery can take up to 10 years [19, 20], and normalisation of BMD can be variable and incomplete, with a significant proportion of patients remaining in the osteopenic/osteoporotic range [20]. This results in a prolonged heightened fracture risk exposure despite remission of hypercortisolism [21].

Unfortunately, specific guidelines for managing bone health in endogenous CS are lacking. Existing recommendations are largely adapted from guidelines for exogenous CS [22]. These recommend regular fracture risk assessment using the Fracture Risk Assessment Tool (FRAX), vitamin D measurement, lumbar spine and hip BMD measurement, and lateral thoracolumbar X‐rays in symptomatic patients or those with low BMD [23]. Moreover, adequate calcium and vitamin D intake should be maintained [24] and bone‐protective agents recommended if exposure to supraphysiological doses of exogenous glucocorticoids is expected for > 3 months [22]. However, quantifying the burden of hypercortisolaemia in endogenous CS is inherently difficult, therefore hindering direct comparisons with the effects of exogenous glucocorticoids on skeletal health. Additionally, the use of bone‐protective agents like bisphosphonates to manage skeletal complications in endogenous CS remains debatable [20, 25].

Given the absence of targeted guidelines and resultant uncertainty in the optimal approach surrounding managing bone health in this condition, we aimed to examine clinicians' engagement with bone health assessment and management in patients diagnosed with endogenous CS using real‐world data. Our findings could support the development of specific evidence‐based guidelines to improve bone health outcomes in this high‐risk population.

## 2 Materials and Methods

### 2.1 Study Design and Subjects

A retrospective study was conducted using Cerner Electronic Health Records of patients diagnosed with endogenous CS due to Cushing's disease, adrenal Cushing's, or ectopic ACTH‐dependent Cushing's at Imperial College Healthcare NHS Trust. Eligible CS patients were aged ≥ 18 years, managed surgically or medically in the last 15 years, and achieved remission from hypercortisolaemia. Patients with cyclical CS, autonomous cortisol secretion (subclinical CS), or CS secondary to adrenocortical carcinoma were excluded due to the heterogeneity of these conditions.

Overt endogenous CS was diagnosed based on clinical features and a combination of the following established diagnostic investigations: overnight/low‐dose dexamethasone suppression test, 24‐h urinary‐free cortisol, and late‐night salivary cortisol and cortisone. Remission was defined by the presence of biochemical hypo/normo‐cortisolaemia following therapeutic intervention. The time to CS diagnosis was calculated as the interval between the reported onset of symptoms and confirmed CS diagnosis.

### 2.2 Data Collection

Data were collected at three time‐points: baseline (time of diagnosis), follow‐up 1 (≤ 1 year after achieving remission), and follow‐up 2 (> 1 year after achieving remission). If multiple datasets were available for follow‐up 2, the most recent assessments were used.

Biochemical markers such as adjusted calcium, phosphate, alkaline phosphatase (ALP), parathyroid hormone (PTH), and vitamin D were recorded. These biochemical parameters were assayed using the standard Abbott Alinity platform.

Data collected on the management of bone health included the use of calcium and/or vitamin D supplementation or optimisation of dietary calcium intake, and/or the initiation and timing of bone‐protective agents such as bisphosphonates, denosumab, or teriparatide.

Available DEXA scans (performed on a GE Lunar Prodigy scanner) were reviewed, and the following data were collected: absolute BMD in g/cm^2^ [2], *T*‐scores, and *Z*‐scores measured at the lumbar spine (L2‐L4), total hip, and femoral neck. The World Health Organisation (WHO) criteria were applied to categorise normal mineralisation, osteopenia, and osteoporosis. *Z*‐scores ≤ −2.0 SD were defined as “below the expected range for age, sex, and ethnicity [26].”

For fracture data, medical notes and radiology reports were reviewed to identify any history of fractures. Fractures were classified as fragility (fall from standing height or less) or traumatic based on mechanism. Baseline fractures occurring within the 2 years preceding endogenous CS diagnosis, were identified [13, 18]. Multiple fractures noted at one time‐point (e.g., various vertebral fractures) were counted as a single fracture event.

Data was collected on the date(s) and type of surgical or medical treatment of hypercortisolism, as well as the types, doses, and duration of post‐operative glucocorticoid replacement.

### 2.3 Study Outcomes

The aim of the study was to assess clinicians' engagement with bone health assessment and management in endogenous CS. The co‐primary outcomes to address this aim were:

1. Assessment: Vitamin D evaluation at diagnosis
2. Management: Calcium and/or vitamin D optimisation, including dietary calcium intake, and/or bone‐protective agents at diagnosis as a composite outcome.

Secondary outcomes included changes in BMD over time, including a comparison between individuals treated or not treated with bone‐protective agents and the prevalence of fragility fractures prior to and following diagnosis and during follow‐up.

### 2.4 Statistical Analysis

Statistical analyses were performed using GraphPad Prism software, version 10.2.0. Continuous variables were expressed as mean ± SD, and normality was assessed using the Shapiro–Wilk test. Categorical variables were compared using Fisher's exact test. Continuous variables were compared using two‐tailed unpaired Student's *t*‐test. A mixed‐effects analysis with Tukey's multiple comparisons post hoc test was used to compare changes in absolute BMD and *Z*‐scores across the three time‐points. A subgroup analysis using mixed‐effects analysis with Šídák's post hoc test, compared changes in BMD between baseline and follow‐up, stratified by whether individuals with endogenous CS were treated with bone‐protective agents. A *p* < 0.05 denoted statistical significance.

## 3 Results

### 3.1 Baseline Characteristics

Data were collected from 79 patients diagnosed with endogenous CS, comprising 64 with Cushing's disease, 13 with adrenal Cushing's, and 2 with ectopic ACTH syndrome. Most cases of endogenous CS were managed surgically via trans‐sphenoidal surgery (*n* = 54), adrenalectomy (*n* = 15), both trans‐sphenoidal surgery and adrenalectomy (*n* = 6), and lung resection (*n* = 2). Two patients were receiving medical treatment with osilodrostat while awaiting definitive treatment. Postoperative glucocorticoid replacement therapy was initiated in 69 patients (87.3%), of whom 36 (52.2%) still required glucocorticoid replacement by follow‐up 2. Among these, 25 patients were taking oral prednisolone (mean daily dose of 3.52 ± 1.33 mg), and 10 patients were taking oral hydrocortisone (mean daily dose of 24.25 ± 11.06 mg).

Baseline characteristics are summarised in Table 1. CS diagnosis occurred at a mean age of 42.6 ± 14.5 years, predominantly in females (81%), of whom 25% were post‐menopausal. The mean estimated time to CS diagnosis from symptom onset was 30.6 months. As expected, diabetes mellitus and hypertension were common at diagnosis, with a prevalence of 35% and 61%, respectively, and the mean body mass index (BMI) across the cohort was 32.3 ± 7.8 kg/m^2^ [2]. Regarding other risk factors for fragility fractures, approximately 1% of the cohort had a parental history of hip fractures, 14% were smokers, and 18% consumed over 3 units of alcohol per day. None had prior exposure to prednisolone or an underlying diagnosis of rheumatoid arthritis.

**Table 1:** Baseline characteristics of included patients with endogenous Cushing's syndrome (*N* = 79) and categorised by the aetiology of hypercortisolism.

	All patients (*n* = 79)	CD (*n* = 64)	ACS (*n* = 13)	EAS (*n* = 2)	*p* value^a^
Age (years)	42.6 ± 14.5	41.6 ± 14	45.1 ± 16.7	57 ± 5.7	0.47
Female (%)	64 (81%)	52 (81%)	10 (77%)	2 (100%)	0.71
Postmenopausal female (%)	20 (25%)	15 (23%)	3 (23%)	2 (100%)	> 0.99
Estimated time to diagnosis (months)	30.6 ± 30.6	31.3 ± 32.3	31.4 ± 28.4	15.0 ± 12.7	0.99
Weight (kg)	86.9 ± 22.7	87.8 ± 23.8	82.5 ± 18.1	88 ± 20.5	0.45
BMI (kg/m²)	32.3 ± 7.8	32.7 ± 8.1	30.2 ± 6.2	33.3 ± 9.5	0.29
DM	28 (35%)	22 (34%)	5 (38%)	1 (50%)	0.76
HTN	48 (61%)	37 (58%)	9 (69%)	2 (100%)	0.54
Smokers	14 (18%)	12 (19%)	2 (15%)	0	> 0.99
Alcohol intake > 3 units/day	11 (14%)	11 (14%)	0	0	> 0.99
Parenteral hx of hip fracture	1 (1%)	1 (1%)	0	0	> 0.99
Vitamin D (nmol/L) (RR *50–150)*	52.5 ± 27.8	50.0 ± 27.6	57.7 ± 26.0	95	0.57
Adj calcium (mmol/L) (RR *2.15–2.6)*	2.4 ± 0.1	2.4 ± 0.1	2.4 ± 0.1	2.5	0.54
Phosphate (mmol/L) (RR *0.8–1.4)*	1.1 ± 0.2	1.1 ± 0.2	1.1 ± 0.1	0.84	0.80
ALP (U/L) (RR *30–130)*	83.8 ± 31.9	86.3 ± 32.7	75.8 ± 28.0	58.5 ± 10.6	0.31
PTH (pmol/L) (RR 1.1−6.8)	6.7 ± 3.2	6.2 ± 3.3	6.8 ± 2.0	12.9	0.71
UFC (nmol/24 h)	1065 ± 1180	937.8 ± 859.9	1462 ± 2046	1474	0.53
*Lumbar (L2‐L4) *					
BMD (g/cm²)	1.07 ± 0.19	1.06 ± 0.19	1.12 ± 0.20	1.15	0.46
*T*‐score	−1.24 ± 1.57	−1.39 ± 1.61	−0.55 ± 1.48	−1.2	0.25
*Z*‐score	−0.88 ± 1.82	−1.13 ± 1.83	−0.03 ± 1.64	0.8	0.19
*Total hip *					
BMD (g/cm²)	0.94 ± 0.14	0.93 ± 0.12	0.96 ± 0.20	1.05	0.54
*T*‐score	−0.65 ± 1.11	−0.79 ± 1.04	−0.22 ± 1.45	0.4	0.26
*Z*‐score	−0.63 ± 1.14	−0.82 ± 1.06	−0.05 ± 1.29	0.8	0.13
*Femoral neck *					
BMD (g/cm²)	0.90 ± 0.14	0.90 ± 0.12	0.87 ± 0.22	0.85	0.59
*T*‐score	−0.76 ± 1.04	−0.75 ± 0.97	−0.82 ± 1.47	−0.7	0.89
*Z*‐score	−0.59 ± 1.00	−0.66 ± 1.01	−0.40 ± 1.09	−0.1	0.58
Osteoporosis (%)	8/35 (22.9%)	7/28 (25%)	1/6 (16.7%)	0/1 (0%)	>0.99

*Note:* Data displayed as mean ± SD unless indicated otherwise.

Abbreviations: ACS, Adrenal Cushing's Syndrome; Adj, adjusted; ALP, alkaline phosphatase; BMD, bone mineral density; BMI, body mass index; CD, Cushing's Disease; DM, diabetes mellitus; EAS, Ectopic ACTH Syndrome; HTN, hypertension; PTH, parathyroid hormone; RR, reference range; UFC, urinary free cortisolclats.

^a^ CD versus ACS, (unpaired two‐tailed *t* test for continuous data or Fisher two‐tailed exact probability test for categorical data);

Osteoporosis defined as *T*‐score in either Lumbar spine (L2−L4), Total hip or Femoral neck ≤ −2.5.

The mean lumbar *T*‐scores for the cohort were within the osteopenic range. There was no significant difference in BMD at the lumbar spine, total hip, and femoral neck observed between those diagnosed with Cushing's disease or adrenal Cushing's (*p* = 0.46, 0.54, 0.59, respectively). Osteoporosis was common during active CS, with a prevalence of 22.9% across the cohort.

We found no difference in hypercortisolaemia, assessed by urinary free cortisol (UFC) levels, between pituitary and adrenal CS. There was no significant correlation between baseline UFC levels or time to diagnosis and BMD at any of the skeletal sites (see Table S1).

### 3.2 Bone Health Assessment and Management

Vitamin D levels were measured in only 43% (34/79) of the patients at CS diagnosis (primary outcome), and only 54.4% of the cohort had a vitamin D level checked during a mean follow‐up of 58.8 months (Table 2). By comparison, adjusted calcium, phosphate, and ALP were more frequently measured as part of routine bone profiles at diagnosis (78.4% or higher) and during follow‐up (62% or higher). However, PTH measurements were infrequent, occurring in only 25.3% of patients at baseline and at least 16.5% during follow‐up.

**Table 2:** Proportion of cohort who had vitamin D (primary outcome) or other bone‐related biochemistry measured before and after achieving remission of hypercortisolaemia in endogenous Cushing's syndrome (Total *N* = 79*)*.

	Baseline	Follow‐up 1ª	Follow‐up 2ᵇ
Vitamin D (nmol/L)	34 (43.0%)	38 (48.1%)	43 (54.4%)
Adjusted calcium (mmol/L)	62 (78.4%)	58 (73.4%)	49 (62.0%)
Phosphate (mmol/L)	62 (78.4%)	58 (73.4%)	49 (62.0%)
PTH (pmol/L)	20 (25.3%)	13 (16.5%)	19 (24.1%)
ALP (U/L)	74 (93.7%)	59 (74.5%)	51 (64.5%)

Abbreviations: ALP, alkaline phosphatase; PTH, parathyroid hormone.

^a^ Follow‐up 1: up to one year after achieving remission from hypercortisolaemia (mean duration = 4.8 months);

^b^ Follow‐up 2: over one year after achieving remission from hypercortisolaemia (mean duration = 58.8 months).

At CS diagnosis and when measured, adjusted calcium, phosphate, and ALP levels were within the reference ranges, vitamin D levels were adequate, but towards the lower end of the reference range (52.5 ± 27.8 nmol/L), and PTH levels were at the upper limit of the reference range (6.7 ± 3.2 pmol/L), in those who were tested.

At baseline, only 39.2% (31/79) received bone health management, as the composite outcome of calcium and/or vitamin D optimisation and/or initiation of bone‐protective agents (Table 3). Although this improved at follow‐up, the majority remained without adequate bone health management. Specifically, at baseline, only 27.8% (22/79) of the cohort received calcium supplementation, and 37.8% (30/79) received vitamin D supplementation. Vitamin D use increased at follow‐up to 46.8%. Bone‐protective agents were initiated in only 7.6% (6/79) of patients at baseline.

**Table 3:** Number and proportion of endogenous Cushing's syndrome patients (*N* = 79) receiving bone health management, including those with fractures.

		Baseline	Follow‐up 1	Follow‐up 2
All patients (*N* = 79)	Calcium or vitamin D supplementation or bone‐protective agent	31 (39.2%)	39 (49.4%)	38 (48.1%)
	Calcium supplementation (%)	22 (27.8%)	22 (27.8%)	23 (29.1%)
	Vitamin D supplementation (%)	30 (37.8%)	39 (49.4%)	37 (46.8%)
	Bone‐protective agents (%)	6 (7.6%)	7 (8.9%)	8 (10.1%)
Patients with fracture (treated/total)	Calcium or vitamin D supplementation or bone‐protective agent	10/14 (71.4%)	11/15 (73.3%)	13/19 (68.4%)
	Calcium supplementation (%)	7/14 (50.0%)	9/15 (60.0%)	9/19 (47.4%)
	Vitamin D supplementation (%)	10/14 (71.4%)	11/15 (73.3%)	13/19 (68.4%)
	Bone‐protective agents (%)	3/14 (21.4%)	6/15 (40.0%)	5/19 (26.3%)

CS patients with fractures were more likely to be administered bone health assessment and management, with 71.8%, albeit not all, receiving calcium, vitamin D, or bone‐protective agents. Bone‐protective agents were initiated in only 21.4% of patients with fractures at baseline, although this increased to 40% and 26.3% at follow‐up 1 and 2, respectively.

Commonly used bone‐protective agents were alendronate, followed by intravenous zoledronate and denosumab. One patient with multiple vertebral fractures received teriparatide.

### 3.3 Changes in Bone Densitometry

**Figure 1:**
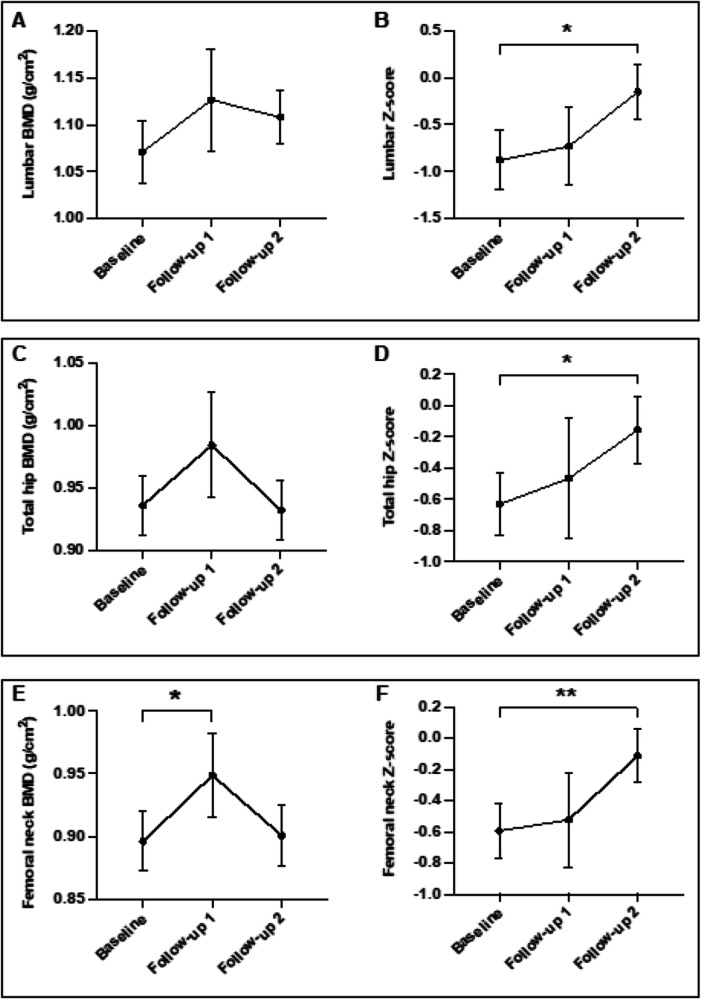
Changes in absolute BMD (g/cm^2^) and *Z*‐scores respectively at (A and B) the lumbar spine, (C and D) total hip, and (E and F) femoral neck before and after achieving remission of hypercortisolaemia in endogenous Cushing's syndrome. Data expressed as mean ± SEM; *p < 0.05; **p < 0.01; mixed‐effects analysis with Tukey's multiple comparisons post‐hoc test applied. Follow‐up 1: up to 1 year after achieving remission from hypercortisolaemia (mean duration = 8.1 months); Follow‐up 2: over 1 year after achieving remission from hypercortisolaemia (mean duration = 66.2 months). *Z*‐score defined as an individual's BMD compared with the average BMD of an age, sex, and ethnicity‐matched control group. BMD, bone mineral density.

Figure 1 shows improvement in BMD following treatment of CS. Notably, femoral neck BMD increased significantly, and a trend towards increased lumbar spine and total hip BMD was observed within 1 year after CS treatment. However, by follow‐up 2, BMD at all three regions showed a downward trend compared to follow‐up 1. By contrast, *Z*‐scores at identical sites progressively increased in a time‐dependent manner after CS remission and were significantly improved by follow‐up 2 compared to baseline. This is explained by the fact that *Z*‐scores are age‐matched, so can increase despite small drops in BMD if less than the expected drop for age.

Accordingly, following remission of CS, the prevalence of osteoporosis (assessed by *T*‐scores) and low bone density for age and sex (assessed by *Z* scores) consistently declined (Table S2). During active CS, only 44.3% (35/79) of the cohort underwent DEXA scans. By follow‐up 2, an additional 41 scans had been performed or repeated, totalling 76 scans throughout the study.

### 3.4 Prevalence of Fractures

**Figure 2:**
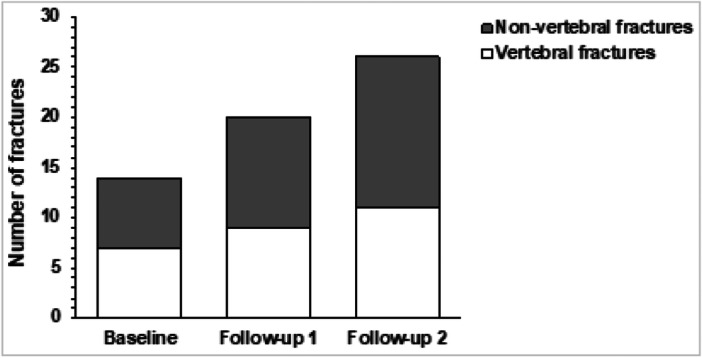
Prevalence of fractures before and after achieving remission of hypercortisolaemia in endogenous Cushing's syndrome, categorised by vertebral and non‐vertebral fractures. Follow‐up 1: up to one year after achieving remission from hypercortisolaemia (mean duration = 6.2 months); Follow‐up 2: over one year after achieving remission from hypercortisolaemia (mean duration = 39.8 months).

Fractures were sustained in 17.7% (14/79) of the cohort within 2 years, leading to a diagnosis of CS (Figure 2). Of these, 57.1% (8/14) sustained multiple fractures. Vertebral fractures were the most common fracture site, occurring in 9% (7/79) of patients, with over half sustaining multi‐level vertebral fractures.

New fragility fractures were observed in 7.6% (6/79) of patients at follow‐up 1, including two vertebral fractures. Importantly, 83% (5/6) of these patients had previously experienced fractures at baseline. By follow‐up 2, another 7.6% (6/79) of patients had new fractures, including two vertebral fractures.

Overall, 26 total fragility fracture events were recorded from baseline, across a mean follow‐up period of 39.8 months. Of these, almost half of the fractures (46.2%) occurred during follow‐up, despite the remission of hypercortisolism, highlighting ongoing fracture risk.

### 3.5 Use of Bone‐Protective Agents

**Figure 3:**
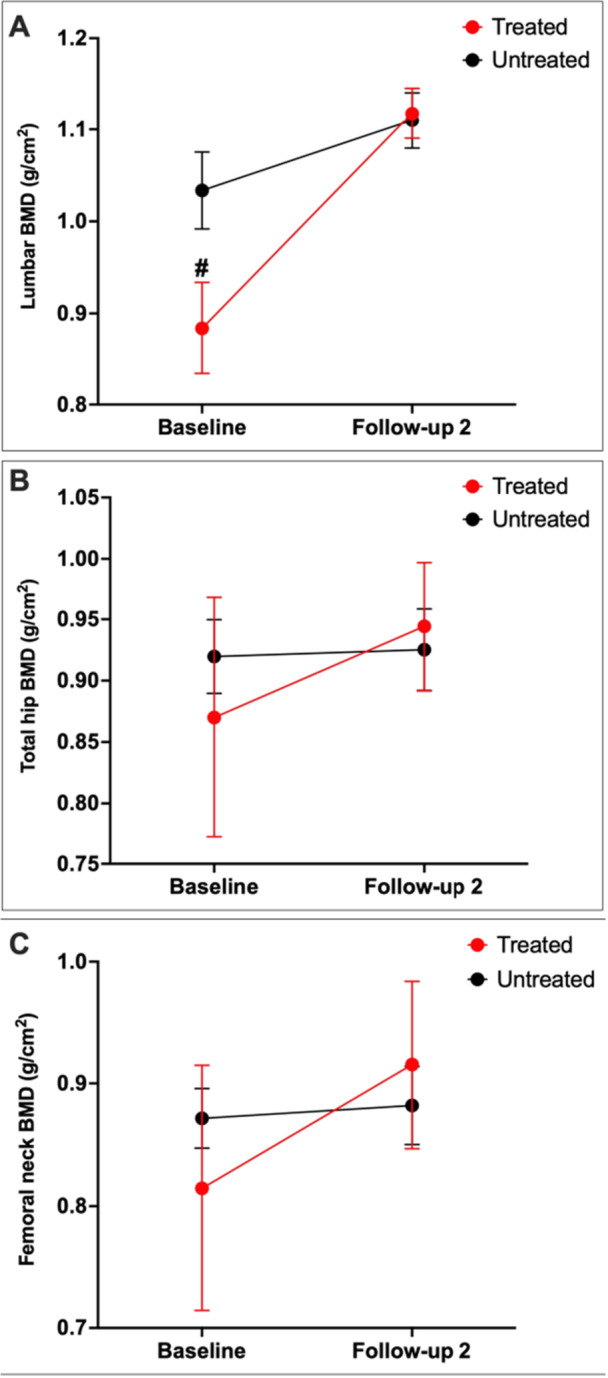
Changes in absolute BMD at the lumbar spine (A), total hip (B), and femoral neck (C) in people with endogenous Cushing's Syndrome who were treated (*n* = 6–8) or not treated (*n* = 17) with bone‐protective agents. Data presented as mean ± SEM. A mixed‐effects analysis with Šídák's post hoc test was applied. #*p* < 0.05 BMD between patients treated and not treated with bone‐protective agents at baseline; Follow‐up 2: over one year after achieving remission from hypercortisolaemia (mean duration = 66.2 months). BMD, bone mineral density.

A subgroup analysis compared changes in BMD from baseline to follow‐up 2 (mean duration: 66.2 months) between patients treated with bone‐protective agents with those who did not (Figure 3). At baseline, lumbar spine BMD was significantly lower in those treated with bone‐protective agents compared to those not treated, which likely contributed to the decision to treat.

Despite starting at a lower BMD, those who received bone‐protective agents showed a significant improvement in lumbar spine BMD, ultimately reaching levels comparable to the untreated group by follow‐up 2. Changes in total hip and femoral neck BMD were not significantly different between these groups (*p* = 0.46 and 0.07, respectively).

## 4 Discussion

Skeletal fragility is an under‐recognised and often neglected complication of endogenous CS [3]. For the first time, clinicians' engagement with bone health assessment and management in endogenous CS was evaluated using real‐world data from an endocrine tertiary centre.

Baseline bone health assessment, as determined by serum vitamin D measurement, was performed in only 43% of patients. This is concerning since active CS is known to affect vitamin D metabolism, impairing the conversion of cholecalciferol into 25‐hydroxy vitamin D, and reducing the expression of vitamin D receptors in peripheral tissues [27]. This increases the risk of vitamin D deficiency compared to healthy controls [4]. Accordingly, vitamin D levels were at the lower limit of the reference range during active CS. Additionally, the mean BMI for this cohort was 32.3 kg/m^2^ [2], with obesity associated with vitamin D insufficiency due to sequestration of vitamin D within adipose tissue [28]. Consequently, the already compromised calcium mobilisation, due to decreased gut absorption and increased urinary excretion, can be further exacerbated, resulting in secondary hyperparathyroidism [27], bone demineralisation, and ultimately fractures. However, it is important to recognise that Vitamin D, as the primary marker of bone health assessment in this study, may not fully capture the complexity of skeletal status; but it represents a pragmatic and specific parameter, signalling active clinical engagement and enabling evidence‐based intervention with proven benefits for bone health.

Despite the lack of targeted guidelines for managing bone health in endogenous CS, it is generally recommended that all patients receive at least calcium and vitamin D supplementation [24]. This recommendation is supported by meta‐analyses demonstrating a mitigating effect on bone loss when combined calcium and vitamin D are used [29]. However, this simple but effective treatment was insufficiently implemented in this high‐risk population. Indeed, bone health was managed in only 39.2% of patients with active CS using calcium and/or vitamin D optimisation, including dietary calcium intake and/or bone‐protective agents. Patients with a fracture at baseline were more likely to receive bone health management (71.4%), but not all did.

DEXA scans were performed in only 44.3% of patients, and less than a third of the cohort underwent a DEXA scan during follow‐up. Nonetheless, skeletal impairment was highly prevalent, with 22.9% of patients fulfilling the criteria for osteoporosis at diagnosis. This is in accordance with the prevalence of osteoporosis reported as between 22% and 57% in patients with active endogenous CS [2]. Moreover, BMD was lowest at the lumbar spine, in keeping with hypercortisolaemia preferential targeting of trabecular over cortical bone [8]. Also, consistent with previous studies, no significant difference was found in the prevalence of osteoporosis and lumbar spine or hip BMD between people with Cushing's disease and adrenal Cushing's [9, 10].

Our cohort included several recognised additional risk factors for low BMD. Although predominantly female, only a third were post‐menopausal, and a similar proportion had type 2 diabetes, suggesting that hypercortisolaemia was the primary driver of bone loss. While obesity is typically associated with higher bone mass, in CS, it may contribute to skeletal fragility due to adverse effects on bone quality.

Endogenous CS tends to run an insidious course, with a mean time to diagnosis of 30.6 months in our study. A comparable diagnostic interval of 34 months has been recorded in a previous meta‐analysis [30]. The diagnostic delay, coupled with a high prevalence of skeletal impairment, highlights the need for greater recognition of the sequelae of CS on bone and calls for timely intervention.

We found no significant correlation between the severity of hypercortisolaemia (based on UFC levels) or time to diagnosis (a proxy for temporal high cortisol exposure) with BMD. This may reflect limited data availability, as not all participants underwent DEXA. It may also indicate that BMD is relatively preserved in this younger cohort, with impaired bone quality, rather than density, contributing more to fragility. Further study is needed to explore this.

However, we found that treatment of hypercortisolism progressively improved bone mineralisation. Even though absolute lumbar and hip BMD decreased at follow‐up 2 after an initial improvement, *Z*‐scores at all skeletal sites consistently increased after the remission of CS. The long‐term reduction in absolute BMD is consistent with physiological bone loss associated with ageing. However, this decline was less pronounced when compared to a matched cohort for age, sex, and ethnicity, accounting for the persistent increase in Z‐scores over time. These findings align with previous studies where lasting improvements in BMD were observed following CS remission [14, 15, 17, 18].

Bone‐protective agents were initiated in only 7.6% of patients. Bisphosphonates, including oral alendronic acid or intravenous zoledronic acid, were most frequently used. Subcutaneous denosumab was often administered in cases of bisphosphonate intolerance. This treatment approach aligns with the use of bisphosphonates as the first‐line agents to manage skeletal complications in exogenous CS [22]. However, the use of bone‐protective agents in endogenous CS is controversial. Randazzo et al. demonstrated significant but comparable bone recovery as early as 2 years after CS remission in patients treated with or without bisphosphates [20]; although these findings are limited by the small sample size. Conversely, Di Somna et al. [25] reported that 12 months of bisphosphonate treatment induced a sixfold greater improvement in lumbar spine BMD compared to cortisol normalisation alone. However, this study used ketoconazole to normalise cortisol levels—an approach which has previously been shown to reduce BMD recovery, potentially by interfering with activation of vitamin D [31].

In the sub‐group analysis, as expected, patients treated with bone‐protective agents started with lower baseline BMD compared to their counterparts, likely reflecting a greater burden of hypercortisolaemia as the primary driver of bone fragility. Regardless, lumbar spine BMD improved markedly in patients receiving bone‐protective agents, with recovery up to the BMD of those not treated. This supports the use of bone‐protective agents to accelerate skeletal recovery after CS treatment [25], especially in people with the highest risk of skeletal complications [24]. However, concerns exist regarding the long‐term suppressive effects of anti‐resorptives on bone turnover, which potentially could hinder bone modelling and recovery post‐CS remission [24]. Therefore, teriparatide, an osteoanabolic agent, could theoretically be preferred to promote skeletal recovery [32, 33].

Fractures occurred in 17.7% of patients, all within the 2 years prior to a diagnosis of CS. This is consistent with previous studies reporting fracture prevalence between 19% and 21% [14, 34], with the highest risk occurring 2‐3 years before CS diagnosis [12, 13]. The diagnostic delay of up to 3 years, which is typical of endogenous CS, creates a critical window of heightened fracture risk due to uncontrolled hypercortisolaemia.

Over half of the fractures at baseline were vertebral fractures, affecting 9% of patients. Higher fracture rates of between 15% and 76% have been reported in other studies [9, 10, 35]. This disparity likely stems from differing methodologies. Tauchmenova et al. [9] routinely imaged the thoracolumbar spine, thereby identifying asymptomatic vertebral fractures, which were present in 48% of cases [9]. However, in our study, spinal radiographs were performed only in symptomatic patients, which likely resulted in a conservative estimate of vertebral fracture prevalence.

Interestingly, fractures occurred despite the restoration of normocortisolaemia and improvements in BMD. Twelve new fractures occurred during a mean follow‐up time of 39.8 months, with five individuals experiencing recurrent fractures. This is concerning and consistent with established evidence that a history of prior fragility fractures is a strong predictor of subsequent fractures [13]. A plausible explanation for the ongoing fracture risk despite CS remission is the persistence of impaired bone quality, despite bone remineralisation. This aligns with studies reporting fractures in patients with normal BMD but poor bone microarchitecture [4, 5], similar to conditions such as type 2 diabetes. Additionally, following surgical remission of endogenous CS, some patients may be receiving replacement doses of glucocorticoids exceeding physiological needs. Cumulative glucocorticoid overexposure can impair bone health, reinforcing the need for ongoing bone health monitoring and management in this group.

Strengths of our study include the large cohort of 79 patients, considering the rarity of endogenous CS. Real‐world data were collected from a tertiary centre, reflecting current clinical practice and highlighting gaps in the existing bone guidelines. Furthermore, a comprehensive dataset including clinical, radiological, and biochemical bone health parameters with a longitudinal design, spanning several years from CS diagnosis, was evaluated.

However, this study has several limitations. First, the retrospective study design meant that data such as densitometry results or fracture histories were not always available, and the prevalence of vertebral fractures is likely to be under‐reported. Second, non‐adherence to bone‐protective agents was not assessed but may have influenced the BMD outcomes. Third, findings were based on a single‐centre and therefore limit generalisability. Finally, the use of vitamin D alone as the primary marker of bone health assessment does not constitute a comprehensive evaluation of skeletal status but should be interpreted alongside the biochemical, densitometry, and clinical parameters presented in the study.

The optimal timing and choice of osteoporosis treatment in CS remains an area of clinical uncertainty. However, we advocate for early fracture risk assessment at diagnosis using established tools like FRAX to guide decisions on DEXA scanning and treatment. Ensuring optimisation of calcium and vitamin D status through diet and/or supplementation remains essential [23]. If vitamin D testing is delayed or unavailable, pragmatic supplementation should be initiated. The key priority is prompt remission from CS to reduce the adverse impact of hypercortisolaemia on bone health. After remission, bone density typically improves, but recovery can be slow. In some cases, a nuanced and individualised approach is warranted. In those with very low BMD or existing fractures, pharmacological intervention may be necessary, even prior to CS treatment, especially if delayed. Both antiresorptive and anabolic agents can be considered to accelerate skeletal recovery. However, when viewed through the lens of glucocorticoid‐induced osteoporosis, anabolics appear to offer superior benefits [36]. Choice of bone‐specific treatment should be tailored to factors such as age, reproductive status, fracture history, and expected time to remission.

This study is novel in its focus on clinician practices in assessing and managing bone health in patients with CS, and benefits from one of the largest cohorts with long follow‐up. Despite the high prevalence of skeletal impairment and fractures, bone health was assessed in only 43% and actively managed in 39.2% of patients. Although BMD recovery post‐CS remission may offer reassurance, fracture risk persisted, with 12 (46.2%) of fractures occurring within 4 years post‐remission.

In summary, our findings reveal a clear mismatch between high fracture burden and low clinical engagement in bone health assessment in endogenous CS, reinforcing the need for targeted clinical guidelines to standardise assessment and support timely intervention.

## Conflicts of Interest

P.B. has received travel support from Theramex. A.N.C. has received travel support and speaking honoraria from Astellas, UCB, and Amgen outside the submitted work. The other authors declare no conflicts of interest.

## Supporting information


**Supplementary Table 1:** Correlation of Urinary Free Cortisol Levels and Time to Diagnosis with Baseline Bone Mineral Density. **Supplementary Table 2:** Prevalence of skeletal impairment before and after achieving remission of hypercortisolaemia in endogenous Cushing's syndrome, assessed by T‐scores and Z‐scores.

## References

[1] S. Wengander , P. Trimpou , E. Papakokkinou , and O. Ragnarsson , “The Incidence of Endogenous Cushing's Syndrome in the Modern Era,” Clinical Endocrinology 91, no. 2 (2019): 263–270, 10.1111/cen.14014. 31094003

[2] D. Leszczyńska , A. Szatko , L. Papierska , W. Zgliczyński , and P. Glinicki , “Musculoskeletal Complications of Cushing Syndrome,” Rheumatology 61, no. 4 (2023): 271–282, 10.5114/reum/169889. 37745145 PMC10515123

[3] S. Frara , A. Allora , L. di Filippo , et al., “Osteopathy in Mild Adrenal Cushing's Syndrome and Cushing Disease,” Best Practice & Research Clinical Endocrinology & Metabolism 35, no. 2 (2021): 101515, 10.1016/j.beem.2021.101515. 33795196

[4] B. Stachowska , J. Halupczok‐Żyła , J. Kuliczkowska‐Płaksej , J. Syrycka , and M. Bolanowski , “Decreased Trabecular Bone Score in Patients With Active Endogenous Cushing's Syndrome,” Frontiers in Endocrinology 11 (2021): 593173, 10.3389/fendo.2020.593173. 33584537 PMC7874075

[5] H. Vinolas , V. Grouthier , N. Mehsen‐Cetre , et al., “Assessment of Vertebral Microarchitecture in Overt and Mild Cushing's Syndrome Using Trabecular Bone Score,” Clinical Endocrinology 89, no. 2 (2018): 148–154, 10.1111/cen.13743. 29781519

[6] I. Chiodini , M. Torlontano , V. Carnevale , V. Trischitta , and A. Scillitani , “Skeletal Involvement in Adult Patients With Endogenous Hypercortisolism,” Journal of Endocrinological Investigation 31, no. 3 (2008): 267–276, 10.1007/BF03345601. 18401211

[7] Z. E. Belaya , D. Hans , L. Y. Rozhinskaya , et al., “The Risk Factors for Fractures and Trabecular Bone‐Score Value in Patients With Endogenous Cushing's Syndrome,” Archives of Osteoporosis 10, no. 1 (2015): 44, 10.1007/s11657-015-0244-1. 26608406

[8] C. M. Francucci , P. Pantanetti , G. G. Garrapa , F. Massi , G. Arnaldi , and F. Mantero , “Bone Metabolism and Mass in Women With Cushing's Syndrome and Adrenal Incidentaloma,” Clinical Endocrinology 57, no. 5 (2002): 587–593, 10.1046/j.1365-2265.2002.01602.x. 12390331

[9] L. Tauchmanovà , R. Pivonello , C. Di Somma , et al., “Bone Demineralization and Vertebral Fractures in Endogenous Cortisol Excess: Role of Disease Etiology and Gonadal Status,” Journal of Clinical Endocrinology & Metabolism 91, no. 5 (2006): 1779–1784, 10.1210/jc.2005-0582. 16522701

[10] T. Apaydın and D. G. Yavuz , “Assessment of Non‐Traumatic Vertebral Fractures in Cushing's Syndrome Patients,” Journal of Endocrinological Investigation 44, no. 8 (2021): 1767–1773, 10.1007/s40618-020-01496-y. 33420960

[11] G. Arnaldi , T. Mancini , G. Tirabassi , L. Trementino , and M. Boscaro , “Advances in the Epidemiology, Pathogenesis, and Management of Cushing's Syndrome Complications,” Journal of Endocrinological Investigation 35, no. 4 (2012): 434–448, 10.1007/BF03345431. 22652826

[12] O. M. Dekkers , E. Horváth‐Puhó , J. O. L. Jørgensen , et al., “Multisystem Morbidity and Mortality in Cushing's Syndrome: A Cohort Study,” Journal of Clinical Endocrinology & Metabolism 98, no. 6 (2013): 2277–2284, 10.1210/jc.2012-3582. 23533241

[13] P. Vestergaard , J. Lindholm , J. Jorgensen , et al., “Increased Risk of Osteoporotic Fractures in Patients With Cushing's Syndrome,” European Journal of Endocrinology 146, no. 1 (2002): 51–56, 10.1530/eje.0.1460051. 11751067

[14] L. T. Braun , J. Fazel , S. Zopp , et al., “The Effect of Biochemical Remission on Bone Metabolism in Cushing's Syndrome: A 2‐Year Follow‐Up Study,” Journal of Bone and Mineral Research 35, no. 9 (2020): 1711–1717, 10.1002/jbmr.4033. 32315096

[15] C. Di Somma , R. Pivonello , S. Loche , et al., “Effect of 2 Years of Cortisol Normalization on the Impaired Bone Mass and Turnover in Adolescent and Adult Patients With Cushing's Disease: A Prospective Study,” Clinical Endocrinology 58, no. 3 (2003): 302–308, 10.1046/j.1365-2265.2003.01713.x. 12608935

[16] A. Kawamata , M. Iihara , T. Okamoto , and T. Obara , “Bone Mineral Density Before and After Surgical Cure of Cushing's Syndrome Due to Adrenocortical Adenoma: Prospective Study,” World Journal of Surgery 32, no. 5 (2008): 890–896, 10.1007/s00268-007-9394-7. 18210182

[17] L. Fütő , J. Tőke , A. Patócs , et al., “Skeletal Differences in Bone Mineral Area and Content Before and After Cure of Endogenous Cushing's Syndrome,” Osteoporosis International 19, no. 7 (2008): 941–949, 10.1007/s00198-007-0514-x. 18043854

[18] P. van Houten , R. Netea‐Maier , M. Wagenmakers , S. Roerink , A. Hermus , and A. van de Ven , “Persistent Improvement of Bone Mineral Density Up to 20 Years After Treatment of Cushing's Syndrome,” European Journal of Endocrinology 185, no. 2 (2021): 241–250, 10.1530/EJE-21-0226. 34061774

[19] P. J. Manning , M. C. Evans , and I. R. Reid , “Normal Bone Mineral Density Following Cure of Cushing's Syndrome,” Clinical Endocrinology 36, no. 3 (1992): 229–234, 10.1111/j.1365-2265.1992.tb01437.x. 1563076

[20] M. E. Randazzo , E. Grossrubatscher , P. Dalino Ciaramella , A. Vanzulli , and P. Loli , “Spontaneous Recovery of Bone Mass After Cure of Endogenous Hypercortisolism,” Pituitary 15, no. 2 (2012): 193–201, 10.1007/s11102-011-0306-3. 21476062

[21] A. Faggiano , R. Pivonello , M. Filippella , et al., “Spine Abnormalities and Damage in Patients Cured From Cushing's Disease,” Pituitary 4, no. 3 (2001): 153–161, 10.1023/A:1015362822901. 12138988

[22] L. Buckley , G. Guyatt , H. A. Fink , et al., “2017 American College of Rheumatology Guideline for the Prevention and Treatment of Glucocorticoid‐Induced Osteoporosis,” Arthritis & Rheumatology 69, no. 8 (2017): 1521–1537, 10.1002/art.40137. 28585373

[23] M. Tóth and A. Grossman , “Glucocorticoid‐Induced Osteoporosis: Lessons From Cushing's Syndrome,” Clinical Endocrinology 79, no. 1 (2013): 1–11, 10.1111/cen.12189. 23452135

[24] A. Scillitani , G. Mazziotti , C. Di Somma , et al., “Treatment of Skeletal Impairment in Patients With Endogenous Hypercortisolism: When and How?,” Osteoporosis International 25, no. 2 (2014): 441–446, 10.1007/s00198-013-2588-y. 24311114

[25] C. Di Somma , A. Colao , R. Pivonello , et al., “Effectiveness of Chronic Treatment With Alendronate in the Osteoporosis of Cushing's Disease,” Clinical Endocrinology 48, no. 5 (1998): 655–662, 10.1046/j.1365-2265.1998.00486.x. 9666879

[26] International Society for Clinical Densitometry. 2019 ISCD Official Positions—Adult. 2019, accessed May 10, 2024, https://iscd.org/learn/official-positions/adult-positions.

[27] S. Frara , L. di Filippo , M. Doga , P. Loli , F. F. Casanueva , and A. Giustina , “Novel Approaches to Bone Comorbidity in Cushing's Disease: An Update,” Pituitary 25, no. 5 (2022): 754–759, 10.1007/s11102-022-01252-w. 35849272

[28] J. P. Ekwaru , J. D. Zwicker , M. F. Holick , E. Giovannucci , and P. J. Veugelers , “The Importance of Body Weight for the Dose Response Relationship of Oral Vitamin D Supplementation and Serum 25‐Hydroxyvitamin D in Healthy Volunteers,” PLoS One 9, no. 11 (2014): e111265, 10.1371/journal.pone.0111265. 25372709 PMC4220998

[29] J. Homik , M. E. Suarez‐A., B. Shea , A. Cranney , G. A. Wells , and P. Tugwell , “Calcium and Vitamin D for Corticosteroid‐Induced Osteoporosis,” Cochrane Database of Systematic Reviews 2010, no. 7 (2000): CD000952, 10.1002/14651858.CD000952. PMC7046131 10796394

[30] G. Rubinstein , A. Osswald , E. Hoster , et al., “Time to Diagnosis in Cushing's Syndrome: A Meta‐Analysis Based on 5367 Patients,” Journal of Clinical Endocrinology & Metabolism 105, no. 3 (2020): e12–e22, 10.1210/clinem/dgz136. 31665382

[31] G. Luisetto , M. Zangari , V. Camozzi , M. Boscaro , N. Sonino , and F. Fallo , “Recovery of Bone Mineral Density After Surgical Cure, But Not by Ketoconazole Treatment, in Cushing's Syndrome,” Osteoporosis International 12, no. 11 (2001): 956–960, 10.1007/s001980170025. 11804023

[32] S. Y. Kim , O. Davydov , D. Hans , and R. Bockman , “Insights on Accelerated Skeletal Repair in Cushing's Disease,” Bone Reports 2, no. C (2015): 32–35, 10.1016/j.bonr.2015.03.001. 28377951 PMC5365170

[33] J. Y. Han , J. Lee , G. E. Kim , et al., “A Case of Cushing Syndrome Diagnosed by Recurrent Pathologic Fractures in a Young Woman,” Journal of Bone Metabolism 19, no. 2 (2012): 153–158, 10.11005/jbm.2012.19.2.153. 24524047 PMC3780926

[34] N. Ohmori , K. Nomura , K. Ohmori , Y. Kato , T. Itoh , and K. Takano , “Osteoporosis is More Prevalent in Adrenal Than in Pituitary Cushing's Syndrome,” Endocrine Journal 50, no. 1 (2003): 1–7, 10.1507/endocrj.50.1. 12733704

[35] L. Trementino , G. Appolloni , L. Ceccoli , et al., “Bone Complications in Patients With Cushing's Syndrome: Looking for Clinical, Biochemical, and Genetic Determinants,” Osteoporosis International 25, no. 3 (2014): 913–921, 10.1007/s00198-013-2520-5. 24126765

[36] A. D. Taylor and K. G. Sagg , “Anabolics in the Management of Glucocorticoid‐Induced Osteoporosis: An Evidence‐Based Review of Long‐Term Safety, Efficacy and Place in Therapy,” Core Evidence 14 (August 2019): 41–50, 10.2147/CE.S172820. 31692480 PMC6711555

