# Supplementary material for: Clinical Practice Patterns in Bone Health Assessment and Management in Endogenous Cushing's Syndrome
Source: Clin Endocrinol (Oxf). 2025 Dec 21;104(4):302–11. doi: 10.1111/cen.70085 (PMC12954153; doi:10.1111/cen.70085)
Supplement: Supplementary file 1 — Supplementary Table 1: Correlation of Urinary Free Cortisol Levels and Time to Diagnosis with Baseline Bone Mineral Density. Supplementary Table 2: Prevalence of skeletal impairment before and after achieving remission of hypercortisolaemia in endogenous Cushing's syndrome, assessed by T‐scores and Z‐scores. [file CEN-104-302-s001.docx]

**Supplementary Table 1. Correlation of Urinary Free Cortisol Levels and Time to Diagnosis with Baseline Bone Mineral Density.**

|  | **Mean (n=79)** | **Lumbar BMD** | | **Total Hip BMD** | | **Neck of Femur BMD** | |
| --- | --- | --- | --- | --- | --- | --- | --- |
|  |  | r | P- value | r | P-value | r | P-value |
| **UFC (nmol/24 hours)** | 1065 ± 1180 | -0.29 | 0.29 | -0.16 | 0.56 | -0.15 | 0.59 |
| **Time to diagnosis (months)** | 30.6 ± 29.9 | 0.26 | 0.33 | 0.08 | 0.78 | 0.07 | 0.79 |

*Data are expressed as mean ± SD. r: Pearson Corelation Coefficient.*

*Abbreviations: UFC: Urinary Free Cortisol, BMD: Bone Mineral Density*

**Supplementary Table 2.**Prevalence of skeletal impairment before and after achieving remission of hypercortisolaemia in endogenous Cushing’s syndrome, assessed by T-scores and Z-scores

|  |  | **Baseline (N=35)** | **Follow-up 1^a^ (N=17)** | **Follow-up 2^b^ (N=24)** |
| --- | --- | --- | --- | --- |
| **T-score** | Normal | 10 (28.6%) | 7 (41.2%) | 6 (25.0%) |
|  | Osteopenia | 17 (48.6%) | 8 (47.1%) | 17 (70.8%) |
|  | Osteoporosis | 8 (22.9%) | 2 (11.8%) | 1 (4.2%) |
| **Z-score** | Within the expected range for age | 21 (63.6%) | 13 (81.3%) | 21 (91.3%) |
|  | Below the expected range for age | 12 (36.4%) | 3 (18.8%) | 2 (8.7%) |

*Data are expressed as mean ± SD*

*^a^Follow-up 1: up to one year after achieving remission from hypercortisolaemia (mean duration = 8.1 months); ^b^Follow-up 2: > one year after achieving remission from hypercortisolaemia (mean duration = 66.2 months)*
